# Development and Validation of a Mechanistic, Weather-Based Model for Predicting *Puccinia graminis* f. sp. *tritici* Infections and Stem Rust Progress in Wheat

**DOI:** 10.3389/fpls.2022.897680

**Published:** 2022-05-27

**Authors:** Irene Salotti, Federica Bove, Vittorio Rossi

**Affiliations:** ^1^Department of Sustainable Crop Production (DI.PRO.VES.), Università Cattolica del Sacro Cuore, Piacenza, Italy; ^2^Horta Srl, Piacenza, Italy

**Keywords:** epidemiological modelling, model evaluation, black rust, disease onset, disease progress

## Abstract

Stem rust (or black rust) of wheat, caused by *Puccinia graminis* f. sp. *tritici* (*Pgt*), is a re-emerging, major threat to wheat production worldwide. Here, we retrieved, analyzed, and synthetized the available information about *Pgt* to develop a mechanistic, weather-driven model for predicting stem rust epidemics caused by uredospores. The ability of the model to predict the first infections in a season was evaluated using field data collected in three wheat-growing areas of Italy (Emilia-Romagna, Apulia, and Sardinia) from 2016 to 2021. The model showed good accuracy, with a posterior probability to correctly predict infections of 0.78 and a probability that there was no infection when not predicted of 0.96. The model’s ability to predict disease progress during the growing season was also evaluated by using published data obtained from trials in Minnesota, United States, in 1968, 1978, and 1979, and in Pennsylvania, United States, in 1986. Comparison of observed versus predicted data generated a concordance correlation coefficient of 0.96 and an average distance between real data and the fitted line of 0.09. The model could therefore be considered accurate and reliable for predicting epidemics of wheat stem rust and could be tested for its ability to support risk-based control of the disease.

## Introduction

Stem rust, also known as black rust, is caused by *Puccinia graminis* Pers. f. sp. *tritici* Erik. and E. Henn. (*Pgt*), and is a major threat to wheat production worldwide (Leonard and Szabo, 2005; Newcomb et al., 2016; Steffenson et al., 2017). Stem rust is potentially the most damaging of the wheat rusts, because it attacks leaf blades as well as leaf sheaths, stems, and heads (Eversmeyer and Kramer, 2000), and it can destroy an entire crop in a few weeks (Roelfs, 1985; Singh et al., 2006; Steffenson et al., 2017) by causing different kinds of damage (Durbin, 1984; Roelfs, 1985; Willocquet et al., 2021). Known since Roman times, the cyclic occurrence of stem rust was recorded in the first half of the 20th century in Europe, United States, India, and Australia (Zadoks, 1963; Rees, 1972; Nagarajan and Joshi, 1975; Roelfs, 1978, 1977). The importance of the disease decreased between the 1960s and 1990s thanks to the introduction of resistance genes in cultivated wheat and to the eradication of the alternate hosts (*Berberis* spp.; Singh et al., 2015; Saunders et al., 2019). Attention to stem rust has increased since the emergence of the *Pgt* race Ug99 in 1998 in Uganda; Ug99 was not controlled by *Sr31* or other major resistance genes (Pretorius et al., 2000; Singh et al., 2006). Since 2013, outbreaks of wheat stem rust in Europe have been recorded in several countries, and new races have been detected (Shamanin et al., 2016; Bhattacharya, 2017; Saunders et al., 2019; Hovmøller et al., 2021). According to recent studies (Lewis et al., 2018), in addition to the evolution of virulent populations, climate change over the past 25 years may help explain the re-emergence of the disease, because *Pgt* is favored by high temperatures (Leonard and Szabo, 2005).

Losses due to stem rust have typically ranged between 10 and 50% but can exceed 90% when epidemics occur early in the season and are not controlled (Beard et al., 2006). Severe epidemics have recently occurred worldwide. In 2015, more than one million ha of spring wheat in Western Siberia and Kazakhstan were affected by stem rust, causing average yield losses of 20–30% on a regional scale (Shamanin et al., 2016). In 2016, widespread attacks of stem rust were also observed in Sicily (Italy) and Morocco (Bhattacharya, 2017; Randazzo et al., 2019).

Control of stem rust is based on genetic resistance, eradication of alternate hosts, and application of fungicides. Several breeding programs and international efforts are focused on the development and exploitation of new sources of resistance to wheat stem rust (Schumann and Leonard, 2011). Although more than 50 stem rust resistance genes have been cataloged, effectiveness is limited to some *Pgt* races or is low under field conditions (Singh et al., 2006). The eradication of alternate hosts, which support the *Pgt* sexual cycle, has significantly reduced the number of new races and has helped to stabilize *Pgt* populations (Roelfs, 1982; Jin, 2011). Timely application of fungicides can also control stem rust (Beard et al., 2006; Wanyera et al., 2009; Tadesse et al., 2010; Mengesha, 2020). As documented by Tadesse et al. (2010), fungicides applied at 14- or 21-day intervals reduced disease progress, but only weekly sprays ensured complete control. Repeated calendar sprays, however, are uneconomical (Roelfs, 1985). In Australia, for example, one or two sprays are typically applied to control the disease (Beard et al., 2006). In Sicily (Italy), only one fungicide application is economically sustainable, and many growers do not apply any fungicides to protect their crops against rusts (Randazzo et al., 2019). Time of application must also be considered. Fungicide sprays are more effective when applied early in the disease development, and no control or poor control occurs when fungicides are applied later in the season to severely affected plants (Loughman et al., 2005; Beard et al., 2006).

Simulation and predictive models have been developed for improving stem rust management. Simulation models can support strategic decisions, research priorities, and breeding strategies (Savary et al., 2018). A simulation model developed by Meyer et al. (2017) describes airborne dispersal routes of *Pgt* uredospores on regional and continental scales and also considers environmental suitability for infection after spore deposition. Willocquet et al. (2021) recently developed a simulation model for estimating the yield losses caused by stem rust. Predictive models mainly support tactical disease management. Because of the importance of preventing the first infections in a season, empirical models have been developed to predict stem rust outbreaks (Rees, 1972; Nagarajan et al., 1977, 1976; Mulatu et al., 2020). For instance, Nagarajan et al. (1977, 1976) used counts of uredospores in rain samples to predict infections of wheat stem rust 20 days in advance of the time when uredia can be first seen on the crop. The use of spore-trapping data, which would require a wide network of spore sampling sites, was also suggested to predict stem rust epidemics in northeastern Australia (Rees, 1972); the presence of uredospores, however, does not necessarily result in infection (Rees, 1972; Nagarajan et al., 1977, 1976). Mulatu et al. (2020) developed an empirical model for stem rust prediction based on temperature and relative humidity in Ethiopia between 2010 and 2019. This model, however, refers to two bread wheat varieties and has not been validated by comparison with independent field data.

Process-based, weather-driven models have been shown to be more accurate and robust than empirical ones because process-based, weather-driven models are mechanistic, i.e., they consider key biological events that determine the development of epidemics and they consider the related driving variables, i.e., weather conditions (Caffi et al., 2007; Narouei-Khandan et al., 2020). Mechanistic models can be developed both conceptually and mathematically by means of a systematic literature review and meta-analysis of published data (Rossi et al., 2015).

The current study had three objectives: (i) to collect and analyze the available information on stem rust of wheat; (ii) to develop a mechanistic, dynamic, weather-driven model; and (iii) to test the model’s ability to predict the first seasonal infections of *Pgt* and the progress of stem rust epidemics.

## Materials and Methods

### Literature Search

According to the criteria presented by Okoli and Schabram (2010), a systematic literature review was performed to collect data on the life cycle of *Pgt* and on the relationships between *Pgt* and its hosts. Our last literature search was carried out in 2021 in three bibliographical databases: the CAB Abstract database (^1^accessed on February 12), the Scopus database (^2^accessed on February 18), and the Google Scholar database (^3^accessed on February 19). Papers were searched by combining the following keywords: (i) *Puccinia graminis* f. sp. *tritici*; (ii) stem rust of wheat OR black rust of wheat OR other common names; (iii) life cycle OR uredospores OR germination OR penetration OR infection OR incubation OR latency OR survival OR deposition. All of the papers identified through the search were initially screened by title to remove any that were clearly not relevant for the development of the model (e.g., papers regarding other rust species). The papers were then evaluated for relevance. To be considered relevant, the papers had to satisfy the following criteria: (i) the name of the pathogen or the disease appeared in the title, abstract, or the authors’ keywords; (ii) as indicated by the abstract, the paper concerned the biology, ecology, or epidemiology of *Pgt*; and (iii) the paper was published in a journal, proceeding, or other forms (including reports or web-sites) from competent authorities/organizations. Papers not excluded at these levels were screened at the full-text level to ensure relevance. Reference lists in the reviewed papers were screened, and additional papers (in addition to those retrieved in the mentioned databases) fulfilling the inclusion criteria were also retrieved and reviewed.

### Disease Cycle

*Puccinia graminis* f. sp. *tritici* is a heteroecious and macrocyclic rust fungus with five spore stages: basidiospores, pycniospores (spermatia), aeciospores, urediniospores (or uredospores), and teliospores. Each spore stage has a role in the fungus life cycle (Littlefield and Heath, 1979; Littlefield, 1981; Roelfs, 2010). Because the fungus develops without the sexual cycle in major wheat-growing areas (Roelfs, 1985), our model focuses on uredospores, which are the main spores responsible for disease development on wheat. In mild and tropical climates, the pathogen survives on volunteer cereals or other gramineous hosts, producing local inoculum that can lead to severe epidemics during the wheat growing season (Roelfs, 1985). In temperate climates, the pathogen generally does not overwinter locally (Roelfs, 1985), and epidemics are caused by airborne uredospores that originate from warmer areas (Zadoks and Bouwman, 1985; Eversmeyer and Kramer, 2000). Capable of long-distance transport, airborne uredospores (Stakman and Christensen, 1946; Hirst et al., 1967; Zadoks and Bouwman, 1985; Eversmeyer and Kramer, 2000) are scrubbed from the atmosphere and deposited onto plant surfaces by rain (Rowell and Romig, 1966; Nagarajan et al., 1976). Given favorable temperatures and the presence of free water, uredospores on host tissues germinate and form appressoria with penetration pegs in 3 to 6 h (Roelfs, 1985). Symptoms usually become visible on stems and leaves a few days after infection as light-colored spots (Roelfs, 1985) from which uredia gradually erupt as masses of reddish-brown uredospores (Zillinsky, 1983). Sporulation continues over several weeks, until plants approach maturity and tissues senesce (Zillinsky, 1983; Roelfs, 1985; Newton et al., 1999). Uredospores detach from uredia and disperse in a diurnal pattern, with spore dispersal peaking at noon (Hirst, 1953). Most of the uredospores produced are deposited within the local wheat canopy (Roelfs and Martell, 1984; Eversmeyer and Kramer, 1992) and survive for several weeks (Roelfs, 1985; Singh et al., 2002).

### Model Description

#### Model Structure

**FIGURE 1:**
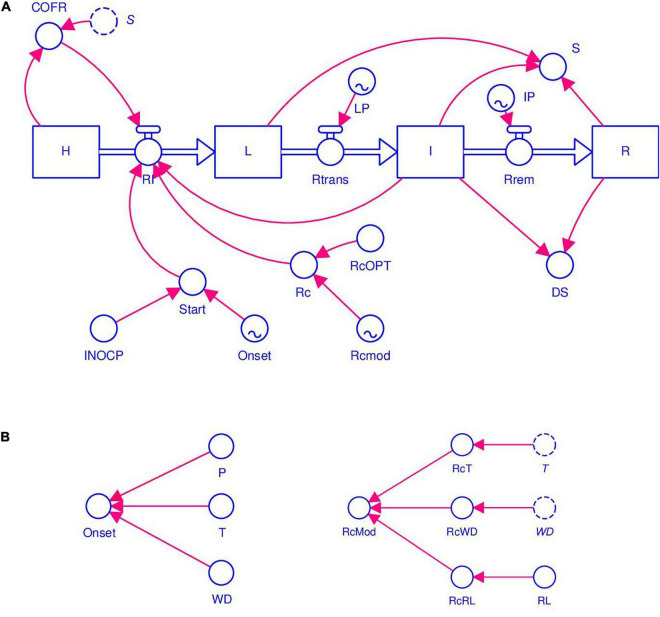
Flow chart of stem rust of wheat. The diagram uses the symbols developed by Forrester (1961). Symbols for state variables, rates, and parameters are listed in Table 1. **(A)** Core structure of the model based on Zadoks (1971), with sites evolving from healthy (HS), to latent (L), to infectious (I), and finally to removed (R). The initiation of an epidemic depends on primary infections (INOCP, Onset). The progress of the epidemic depends on secondary infections (RI, Rc, and I). The model’s core works with a daily time step. **(B)** Conditions for primary infections and modifiers of secondary infections (RcWD, RcT, and RcLR), which are calculated with an hourly time step.

Information retrieved from selected papers was organized based on systems analysis (Leffelaar and Ferrari, 1989) and was used for model development. The model structure is shown in Figure 1. The diagram was drawn following the system representation of Forrester (1997) as used in STELLA^®^ (abbreviation of Systems Thinking, Experimental Learning Laboratory with Animation), a visual programming language for modeling system dynamics. The diagram combines state variables (rectangles), flows (solid arrows), rates (valves), parameters, and coefficients (circles).

**TABLE 1:** List of variables, rates, and parameters used in the model.

Variable type	Acronym	Description	Unit
State variables	H	Healthy sites	0 to 1
	I	Infectious sites, where visible lesions produce uredospores	0 to 1
	L	Latent sites, where stem rust symptoms are not visible	0 to 1
	R	Removed sites, where visible lesions are old and non-sporulating	0 to 1
Rates	RI	Rate of infection	0 to 1
	Rrem	Rate of removals	0 to 1
	Rtrans	Rate of transfers	0 to 1
Parameters	*i*	Duration of infectious period in optimum conditions	N days
	INOCP	Amount of primary infection	0 to 1
	*p*	Duration of latency period in optimum conditions	N days
	*r*	Apparent infection rate	N
	RcOPT	Optimum Rc value	N
	Start	Date of epidemic onset	day
Computed values	COFR	Correction factor for diseased sites	0 to 1
	DS	Disease severity	0 to 1
	Rc	Basic infection rate corrected for the removal	0 to 1
	S	Diseases sites, i.e., S = L + I + R	0 to 1
Climate drivers	LR	Light regime, i.e., absence or presence of light	0/1
	P	Hourly rainfall	mm
	T	Daily or hourly air temperature	°C
	Teq	Equivalent of temperature	0 to 1
	Tmin	Minimum temperature for uredospores germination	°C
	Tmax	Maximum temperature for uredospores germination	°C
	Tw	Mean temperature during a wetness period	°C
	WD	Duration of the wet period	N hours
Driving functions	f(T)_GER_	Equation for the effect of temperature on germination	0 to 1
	f(T)_IP_	Equation for the effect of temperature on infectious period	0 to 1
	f(T)_LP_	Equation for the effect of temperature on latency period	0 to 1
	f(T)_PEN_	Equation for the effect of temperature on penetration	0 to 1
	f(W)_GER_	Equation for the effect of wetness duration on germination	0 to 1
	f(W)_PEN_	Equation for the effect of wetness duration on penetration	0 to 1
	IP	Infectious period	0 to 1
	LP	Latency period	0 to 1
	RcLR	Rc modifier for light regime	0.452/1
	RcT	Rc modifier for temperature	0 to 1
	RcWD	Rc modifier for wetness duration	0 to 1

The core of the model’s structure is based on the epidemiological model developed by Zadoks (1971), in which the crop is thought to consist of a large but finite number of infection sites that have equal dimensions and equal probability of becoming infected. A site is defined as a fraction of the host tissues where an infection may occur and where a lesion may develop (Zadoks, 1971; Savary et al., 2015). A site occurs under one of the following mutually exclusive conditions: (i) heathy (H); (ii) latent (L), i.e., without symptoms of stem rust; (iii) infectious (I), i.e., with visible lesions producing spores; and (iv) removed (R), i.e., lesions are older and non-sporulating (Figure 1 and Table 1). The structure used here represents the host in a very simple manner and does not incorporate host growth or senescence; it also does not account for lesion expansion from infected sites. Sites becoming infected at an infection rate (RI), i.e., they change from healthy sites into latent sites. Latent sites become infectious at the end of a latency period (*LP*) at a transfer rate (Rtrans), and infectious sites become removed at the end of an infectious period (*IP*) with a rate of removal (Rrem) (Figure 1 and Table 1).

At the beginning of model calculations, H = 1 (i.e., the whole crop is healthy), and the model represents the flow from one state to the following one as a proportion of the H, i.e., on a 0 to 1 scale. The model works with a daily time step, with the following exceptions: the onset of primary infection (see Section “Model Structure”) and the modifiers of RI (see Section “Modifiers of RcOPT”) are calculated hourly and are reported on the daily scale.

#### Development and Parameterization of Model Equations

The effects of temperature and wetness duration on epidemics are incorporated into the model as mathematical equations. The effects of temperature on germination, penetration, the length of the latency period, and the length of the infectious period are described by the following equations: f(T)_GER_, f(T)_PEN_, f(T)_LP_, and f(T)_IP_ (see Sections “Modifiers of RcOPT,” “Latency Period,” and “Infectious Period”). The effects of wetness duration on germination and penetration are described by the equations f(WD)_GER_ and f(WD)_PEN_, respectively (see Section “Modifiers of RcOPT”).

Equations were developed and parameterized based on the papers that were collected through the systematic literature review. Useful data were retrieved from tables, figures, and texts of the selected papers. The shape of the data and the Akaike information criterion (Burnham and Anderson, 2002) were used to choose the best-fitting mathematical functions. Equation parameters were estimated using the function *nls* of the “stats” package of R software (Team, R Core. R: A Language and Environment for Statistical Computing. 2019, which is available at^4^). The parameterized equations were evaluated for goodness-of-fit based on the adjusted R^2^, the concordance correlation coefficient (CCC), the root mean square error (RMSE), and the coefficient of residual mass (CRM) (Nash and Sutcliffe, 1970; Lin, 1989). The adjusted R^2^ was estimated by conducting a linear regression between the observed values and the model predicted values; the linear regression was conducted with the *lm* function of the R “stats” package (Wickham, 2019). The CCC is a measure of model accuracy (Madden, 2006), and is calculated as the product of the Pearson correlation coefficient and the Cb coefficient; the CCC indicates the difference between the best fitting line and the perfect agreement line (Lin, 1989). The CCC was obtained using the CCC function of the R “DescTools” package (Signorell, 2020). The RMSE, which represents the average distance of real data from the fitted line (Nash and Sutcliffe, 1970), was obtained using the *rmse* function of the R “modelr” package (Wickham, 2019). The CRM is a measure of the tendency of the equation to overestimate or underestimate the observed values (a negative CRM indicates a tendency of the model toward overestimation) (Nash and Sutcliffe, 1970).

#### First Seasonal Infection

The date of the establishment of the epidemic (Start) depends on Onset, which represents the occurrence of weather conditions favorable for the first infection of the season. Onset is calculated hourly and reported at a daily time step as either Onset = 0, i.e., conditions are not favorable for infection, or Onset = 1, i.e., conditions are favorable for primary infection. Information retrieved from Singh et al. (2006, 2002), Roelfs (1985); Burrage (1970), Rowell and Romig (1966), and Sharp et al. (1958) were used to set Onset = 1 as follows: (i) rain event (P) ≥ 1 mm h^–1^; (ii) the wetness period (WD) following rain is ≥3 h; and (iii) mean temperature during WD (Tw) is between 15 and 32°C. At Onset, the proportion of sites entering L depends on the parameter INOCP, which is an estimate of RI for the first seasonal infection.

#### Secondary Infections

Once the first seasonal infection has established, secondary infections are responsible for the development of epidemics through the transfer of sites from healthy to latent through a rate of infection (RI; i.e., the proportion of newly infected sites per unit of time). RI is modeled as a function of the number of infectious sites (I), a basic infection rate corrected for the removal (Rc; Vanderplank, 1963), and a correction factor for diseased sites (COFR) as follows:


(1) RI=Rc×I×COFR

COFR is calculated as follows:


(2) COFR= 1-(S/(S+H))

where S is the sum of disease sites (S = L + I + R).

In equation (1), Rc represents the proportion of daughter lesions generated per mother lesion. In the model, Rc depends on the optimum corrected basic infection rate (RcOPT), which is the basic infection rate under optimum environmental conditions on a susceptible wheat variety, and on modifiers (RcMod). RcMod is the daily reported value of modifiers for the effect of temperature (RcT), wetness duration (RcWD), and light regime (RcLR) calculated hourly. Rc is calculated as follows:


(3) Rc=RcOPT×RcMod

RcOPT was estimated following Sun and Zeng (1994) from disease progress curves as follows:


(4) $$\mathrm{RcOPT}=\frac{r}{\text{exp}\left(-r\times p\right)-\exp\left(-r\times \left(i+p\right)\right)}$$

where *p* is the latency period, which was set as 9 days under favorable conditions for epidemics (Mortensen and Green, 1978); *i* is the infectious period, which was set as 32 days under favorable conditions for epidemics (Mortensen and Green, 1978); and *r* is the apparent infection rate (Vanderplank, 1963, 1975), calculated as follows:


(5) $$r=\frac{\ln\left(x^{2}/x^{1}\right)}{\left(t^{2}-t^{1}\right)}$$

where x_1_ and x_2_ are disease fractions on two successive dates (t_1_ and t_2_) at the early stage of the epidemic under conditions conducive to the disease. Equation (5) was used to calculate *r* from published disease progress curves in susceptible and unprotected crops (Romig and Calpouzos, 1970; Nazareno and Roelfs, 1981; McGrath and Pennypacker, 1991). The first two non-zero severity values (expressed on a 0 to 1 scale) were retrieved from these curves and used for the calculation, resulting in an RcOPT value of 4.5.

Modifiers of RcOPT are used in the model to account for major environmental conditions influencing the infection process (Loomis and Adams, 1983). As mentioned before, modifier contributions are calculated by the model on an hourly basis and are subsequently reported as RcMod on a daily basis; this enabled the model to accurately account for the effects of environmental conditions on infection. RcMod is calculated as follows:


(6) RcMod=RcT×RcWD×RcLR

#### Modifiers of RcOPT

Development and parameterization of modifiers of RcOPT were based on the available literature on urediospore germination and germ tube penetration *via* stomata.

The modifier RcT accounts for the effect of air temperature (T, in°C) on both germination (f(T)_GER_) and penetration (f(T)_PEN_), as follows:


(7) $$\mathrm{RcT}=f{\left(T\right)}_{\mathrm{GER}}\times f{\left(T\right)}_{\mathrm{PEN}}$$

The effect of T on germination is calculated by a Bete equation (Analytis, 1977) in the following form:


(8) $$f{\left(T\right)}_{\mathrm{GER}}={\left(\alpha \times {\mathrm{Teq}}^{\beta }\times \left(1-\mathrm{Teq}\right)\right)}^{\gamma }$$

where Teq is the equivalent of temperature, calculated as Teq = (Tw – Tmin)/(Tmax – Tmin); Tw is the mean temperature recorded during the wet period; Tmin is the minimum temperature for uredospore germination, i.e., 4°C (Burrage, 1970); and Tmax is the maximum temperature for uredospore germination, i.e., 35°C (Katsuya and Green, 1967). Germination does not occur when Tw < Tmin or Tw > Tmax. Equation (8) was developed and parameterized using the data of Burrage (1970) and Katsuya and Green (1967); estimates and standard errors of equation parameters were α = 4.375 ± 1.016, β = 1.391 ± 0.419, and γ = 0.396 ± 0.201, with adjusted *R*^2^ = 0.916, CCC = 0.962, MRSE = 0.1, and CRM = 0.004.

The effect of T on penetration is described by the Duthie equation (Duthie, 1997) in the following form:


(9) $$f{\left(T\right)}_{\mathrm{PEN}}=\zeta \times \frac{\exp\left(\left(\mathrm{Tw}-\delta \right)\times \eta /\left(\varepsilon +1\right)\right)}{1+\exp\left(\left(\mathrm{Tw}-\delta \right)\times \eta \right))}$$

with ζ = ((ε + 1)/ε) × ε^(1/(ε + 1))^; equation (9) was developed and parameterized using the data of Burrage (1970) and Sharp et al. (1958). Estimates and standard errors of equation parameters were ε = 2.699 ± 1.83, δ = 27.02 ± 1.37, and η = 0.683 ± 0.155, with adjusted *R*^2^ = 0.824, CCC = 0.914, MRSE = 0.139, and CRM = 0.028.

The modifier RcWD accounts for the effect of wetness duration (WD, in h; i.e., cumulative number of h with leaf wetness) on both germination (f ‘(WD)_GER_) and penetration (f ’(WD)_PEN_), as follows:


(10) RcWD=f(WD)GER′×f(WD)PEN′

The effect of WD on germination is calculated as the first derivative (f ’(WD)_GER_) of the following equation:


(11) $$f{\left(\mathrm{WD}\right)}_{\mathrm{GER}}=\frac{1}{1+\iota \times \exp\left(-\kappa \times \mathrm{WD}\right)}$$

which was developed and parameterized by fitting the data from Burrage (1969, 1970), and Eversmeyer and Kramer (1989). Estimates and standard errors of equation parameters were ι = 35.224 ± 3.663 and κ = 2.27 ± 0.618, with adjusted *R*^2^ = 0.971, CCC = 0.982, MRSE = 0.073, and CRM = –0.066.

The effect of WD on penetration is calculated as the first derivative (f ’(WD)_PEN_) of the following equation:


(12) $$f{\left(\mathrm{WD}\right)}_{\mathrm{PEN}}=1-\lambda \times \exp\left(-\mu \times \mathrm{WD}\right)$$

which was developed and parameterized by fitting the data from Burrage (1970). Because germinated uredospores form germ tubes on stomata about 3 h after the beginning of a wetness period (Roelfs, 1985; Singh et al., 2002), there is no penetration and f(WD)pen = 0 when WD < 3 h. Estimates and standard errors of equation parameter were λ = 1.207 ± 0.124 and μ = 0.068 ± 0.017, with adjusted *R*^2^ = 0.951, CCC = 0.978, MRSE = 0.084, and CMR = 0.032.

The RcLR modifier accounts for the light/dark regime during infection. Germination of uredospores is slightly higher in the dark than in daylight (Givan and Bromfield, 1963), but penetration of germ tubes is substantially inhibited in the dark. Therefore, RcLR = 1 in daylight hours and =0.452 in dark hours (Sharp et al., 1958).

#### Latency Period

Latent sites gradually flow from L to I, with a rate of transfer (Rtrans) governed by a temperature-dependent equation accounting for the latency period (LP). The rate of transfer is calculated daily as the first-order derivative of the following Gompertz equation:


(13) $$f{\left(T\right)}_{\mathrm{LP}}=\exp\left(-\nu \times \exp\left(-\rho \times \mathrm{DD}\right)\right)$$

where DD is the degree-days accumulated from the day in which the infection occurred, with 5°C as the minimum temperature (Singh et al., 2002); therefore, when T ≤ 5°C, T = 0; when T > 5°C, T = (T – 5). Equation (13) was developed and parameterized by fitting the data of Katsuya and Green (1967); estimates and standard errors of equation parameters were ν = 2632 ± 255.8 and ρ = 0.07 ± 0.008, with adjusted *R*^2^ = 0.823, CCC = 0.904, MRSE = 0.084, and CMR = 0.032.

#### Infectious Period

Sites I remain infectious for a period (IP), the length of which is determined by temperature. The flow from I to R is therefore governed by a temperature-dependent rate of removal (Rrem). The length of the infectious period is determined by the following logistic equation:


(14) $$f{\left(T\right)}_{\mathrm{IP}}=\frac{1}{1+\varsigma \times \exp\left(-\sigma \times \mathrm{DD}\right)}$$

where DD is the degree-days accumulated from the day of transfer of sites from L to I, with 5°C as the minimum temperature. As a consequence, when T ≤ 5°C, T = 0; when T > 5°C, T = (T – 5). The f(T)_IP_ ranges from 0 to 1, and sites are removed from I when f(T)_IP_ = 1. Equation (14) was developed and parameterized by fitting the data of Mortensen and Green (1978); estimates and standard errors of equation parameters were ς = 23.97 ± 4.15 and σ = 0.012 ± 0.0006, with adjusted *R*^2^ = 0.965, CCC = 0.982, MRSE = 0.063, and CMR = –0.005.

#### Predicted Disease Severity

The model calculates disease severity (DS) during the progress of the epidemic as the sum of the proportion of sites that are carrying visible lesions, i.e., infectious and removed sites, as follows:


(15) DS=I+R

### Model Evaluation

The model was evaluated for its ability to describe real stem rust epidemics. Model evaluation was conducted separately for the first seasonal infection and for disease progress, using independent data (i.e., data not used in model development).

#### First Seasonal Infection

To evaluate the ability of the model to predict the first seasonal infection of stem rust, model outputs were validated against real data obtained from nine experimental wheat fields in Italy, which were established for a range of both durum (*Triticum durum* L., cv. Fuego, Farah, Telemaco, Antalis, Brancaleone, Salgado, Acropolis, RGT Aventadur, Ramirez, Gibraltar, Puro, Platone, Tirex, Teodorico, Don Matteo, and Pigreco) and bread (*T. aestivum* L., cv. Drusilla, Lucilla, Tocayo, Caronte, Nabucco, Bologna, and Nogal) varieties of wheat between 2016 and 2021 (Table 2). All the tested varieties were susceptible to stem rust. All plots (10 m^2^) in each experimental field were inspected at 5- to 10-day intervals for the onset of stem rust symptoms in any variety. Weather data were recorded by meteorological stations (PESSL iMetos 3.3) located <2 km from the experimental fields, except for USS16 and USS17, where the weather station was about 15 km away.

**TABLE 2:** Comparison between predicted and observed first seasonal infection in the experimental sites in Italy (acronyms for locations and years), periods of disease assessment, and corresponding properties of the model.

Acronym	Location^c^	Year	Period of disease assessment	TPP	TNP	FNP	FPP
RAV17^a^	Ravenna, Emilia-Romagna	2017	May 10–May 29	1	1	0	1
RAV18^a^	Ravenna, Emilia-Romagna	2018	May 24–June 11	0	1	1	1
RAV19^a^	Ravenna, Emilia-Romagna	2019	May 23–June 6	1	2	0	0
RAV20^a^	Ravenna, Emilia-Romagna	2020	May 13–June 5	1	3	0	0
RAV21^a^	Ravenna, Emilia-Romagna	2021	May 18–June 15	1	3	0	0
FOG17^a^	Foggia, Apulia	2017	April 26–May 25	1	3	0	0
FOG19^a^	Foggia, Apulia	2019	May 15–May 30	1	2	0	0
USS16^a^	Ussana, Sardinia	2016	May 27–June 5	1	2	0	0
USS17^b^	Ussana, Sardinia	2017	May 27–June 10	0	3	0	0
Total				7	20	1	2
Proportions				0.875	0.909	0.125	0.091

**Prior Probability (P)**			**Posterior Probability (P)**				

*P*(O+) = 0.27			*P*(O + P+) = 0.78		*P*(O + P–) = 0.04		
*P*(O–) = 0.73			*P*(O–P–) = 0.96		*P*(O–P+) = 0.22		

Likelihood ratio (LR)	LR(+) = TPP/FPP = 9.63						

	LR(–) = FNP/TNP = 0.14						
Youden Index	J = TPP–FPP = 0.784						
Overall accuracy	0.9						

*Cases (periods of disease assessment) were classified as follows: TPP, true positive proportion (sensitivity); TNP, true negative proportion (specificity); FNP, false negative proportion; FPP, false positive proportion.*

*^a^Stem rust symptoms were first detected on the last day of disease assessment.*

*^b^No visible stem rust symptoms were detected during the assessment period.*

*^c^Ravenna, Emilia-Romagna: 44°28′56″N 12°10′49″E; Foggia, Apulia: 41°20′55″N 15°34′07″E; Ussana, Sardinia: 39°24′54″N 9°05′59″E.*

The model was operated by using weather data in each year and location starting from 9 days before the beginning of the disease assessment period (Table 2). Disease assessments (cases) were categorized as either 0 (disease symptoms were not observed on wheat plants, O–) or 1 (disease symptoms were observed on wheat plants, O+). Similarly, cases were categorized as either 0 or 1 based on whether the onset of the disease was predicted (P+) or not (P–) by the model. Because first seasonal infections are seldom severe (Prescott et al., 1986), predicted lesions were considered to be visible for a field assessor when 50% of the uredia generated by the first seasonal infection were predicted to erupt.

According to Madden (2006), a Bayesian analysis was used to evaluate the correspondence between model predictions and stem rust symptoms observed in the field. We tested the hypothesis that new stem rust symptoms were observed in the field (O+) on those days when the model predicted lesion appearance (P+), and that no disease symptoms were observed (O–) on those days when the model did not predict lesion appearance (P–). A contingency table (2 × 2) was prepared containing (i) the true positive proportion (TPP or sensitivity), (ii) the true negative proportion (TNP or specificity), (iii) the false positive proportion (FPP), and (iv) the false negative proportion (FNP).

Prior and posterior probabilities of predicting the first seasonal stem rust occurrence based on model output were calculated (Madden, 2006). The prior probabilities of disease to occur *P*(O+) or not to occur *P*(O–) were compared with the following posterior probabilities: (i) the probability of disease occurrence when predicted by the model, *P*(P + O+); (ii) the probability of disease occurrence when not predicted by the model (i.e., missed real infections), *P*(P–O+); (iii) the probability of no disease occurrence when not predicted by the model, *P*(P–O–); and (iv) the probability of no disease occurrence when predicted by the model (i.e., unjustified alarms), *P*(P + O–). Positive and negative likelihood ratios were calculated as LR(+) = TPP/FPP and LR(–) = FNP/TNP, respectively. The diagnostic ability of the model was evaluated by means of the Youden index, calculated as J = TPP – FPP. Overall model accuracy was estimated as the ratio between correct and total predictions.

#### Disease Progress

To validate the ability of the model to predict disease development throughout the season, six disease progress curves were retrieved from the literature. Details of experimental sites used for disease progress validation are summarized in Table 3. Initial infections resulted from naturally occurring airborne inoculum in two epidemics and from inoculum originating from artificially and heavily infected “spreader wheat rows” located near the experimental plots in four epidemics (Table 3).

**TABLE 3:** Experimental sites (acronyms for locations and years), wheat cultivar, period of disease assessment, INOCP (value of primary inoculum used to initialize model calculations), and weather data used to validate model predictions of epidemic development.

Acronym	Location and Year	Cultivar^c^	Period of assessment	T0^d^	T1^e^	INOCP	Weather data
ROS68^a^	Rosemount, MN, United States; 1968 (Romig and Calpouzos, 1970)	Purdue 5481C-1-13-2	weekly; June 14–July 31	June 14	June 21	0.08	Minneapolis Airport (MSP); 30 km
ROS78^b^	Rosemount, MN, United States; 1978 (Nazareno and Roelfs, 1981)	Prelude	weekly; June 29–July 29	June 29	July 8	0.03	Minneapolis Airport (MSP); 30 km
ROS79^b^	Rosemount, MN, United States; 1979 (Nazareno and Roelfs, 1981)	Prelude	4-day intervals; July 12–July 31	July 12	July 16	0.008	Minneapolis Airport (MSP); 30 km
STP78^a^	St. Paul, MN, United States; 1978 (Nazareno and Roelfs, 1981)	Prelude	3- to 5-days intervals; July 4–August 6	July 9	July 16	0.12	Minneapolis Airport (MSP); 35 km
STP79^a^	St. Paul, MN, United States; 1979 (Nazareno and Roelfs, 1981)	Prelude	4-days intervals; July 12–August 3	July 16	July 20	0.27	Minneapolis Airport (MSP); 35 km
PEN86^a^	Rock Springs, PA, United States; 1986 (Nazareno and Roelfs, 1981)	Tyler	5- to 7-days intervals; May 29–July 1	May 29	June 4	0.14	University Park Airport (KUNV); 20 km

*^a^Initial infection resulted from artificially infected wheat spreader rows planted outside the field.*

*^b^Initial infection resulted from naturally occurring airborne inoculum.*

*^c^All cultivars were susceptible cultivars of Triticum aestivum.*

*^d^Date of last disease assessment in which no stem rust symptoms were observed.*

*^e^Date of disease assessment in which stem rust symptoms were first observed.*

The model was operated beginning at 9 days before the day of the last assessment in which no disease was observed. For epidemics caused by artificially inoculated spreader rows, rain was not considered in the calculation of the variable Onset. Values of INOCP used to initialize model runs are reported in Table 3; because of the use of heavily infected spreader rows, the value of INOCP used for epidemics caused by artificial inoculum was about 10 times greater than the value used for epidemics caused by natural inoculum (Table 3). Because there was no information in the literature for calculating INOCP, values were estimated empirically. For each epidemic, the value of INOCP was determined based on a comparison of predicted and observed final disease severity. The value of INOP that resulted in the closest agreement between the final predicted disease severity and the final observed disease severity was used for model runs.

Predicted disease severities (i.e., sum of infectious and removed sites) and observed disease severities were compared. For the evaluation of model performance, RMSE, CRM, and CCC were calculated (Nash and Sutcliffe, 1970; Lin, 1989).

## Results

### Evaluation of First Seasonal Infection

Stem rust symptoms were observed in 8 of the 9 experimental fields; no visible symptoms of the disease were detected at USS17. Model predictions concerning the occurrence of infection (P+) or no infection (P-) and observation of stem rust appearance (O+) or no appearance (O-) in wheat experimental fields for each year and location are summarized in Table 2. Among the total of 30 cases (i.e., disease assessments) that were considered, 8 showed infection and 22 did not. Real infections were correctly predicted in 7 of the 8 cases, meaning that only 1 real infection was missed (Table 2). Unjustified alarms were recorded in 2 of 22 cases (Table 2). Results of the Bayesian analysis (Table 2) showed sensitivity TPP = 0.875 and specificity TNP = 0.909; the one missed infection and the two unjustified alarms led to FNP = 0.125 and FPP = 0.091, respectively.

The model had an overall accuracy of 0.9 and a Youden index J = 0.784 (Table 2). An increase from the prior to posterior probabilities of correctly predicting an infection was observed, with *P*(O+) = 0.27 and *P*(P + O+) = 0.78. At the same time, the probability of not predicting an infection when there was no infection increased from a prior probability *P*(O–) = 0.73 to a posterior probability *P*(P–O–) = 0.96 (Table 2). The effectiveness of the model as a predictor was ensured by the likelihood of a positive prediction LR(+) = 9.63 (larger than 1) and the likelihood of a negative prediction LR(–) = 0.14 (close to 0) (Table 2). These results indicate that the model was able to effectively predict both infection periods and periods in which the disease did not occur.

**FIGURE 2:**
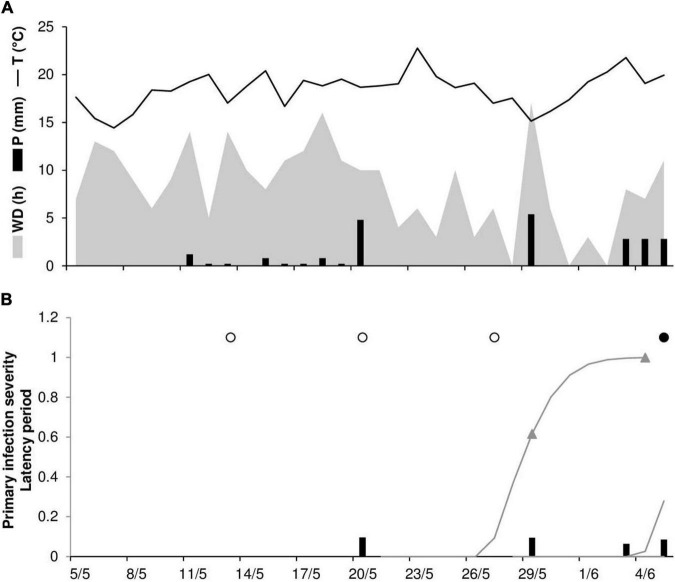
Predicted and observed primary infection onset in the experimental wheat field in Ravenna, Italy, in 2020 (RAV20). **(A)** Weather variables: air temperature (T, °C, solid line), rainfall (R, mm, black bars), and wetness duration (WD, in h, gray area). **(B)** Predicted primary infection (black bars); progress of latency (gray line); period of uredia eruption, from 50 to 100% of erupted uredia (line between triangles); days on which stem rust symptoms were not observed (empty dots); days on which uredia were observed in field plots (full dots).

An example of model output and correct model predictions is shown in Figure 2 for RAV20. Stem rust symptoms were first observed on June 6; no visible lesions were recorded by disease assessments on May 13, 20, or 27. The model predicted the occurrence of the first seasonal infection on May 20 after a rain event of 4.8 mm h^–1^; uredia eruption was predicted to occur between May 28 and June 3, with 50% of uredia eruption predicted to occur on May 29.

**FIGURE 3:**
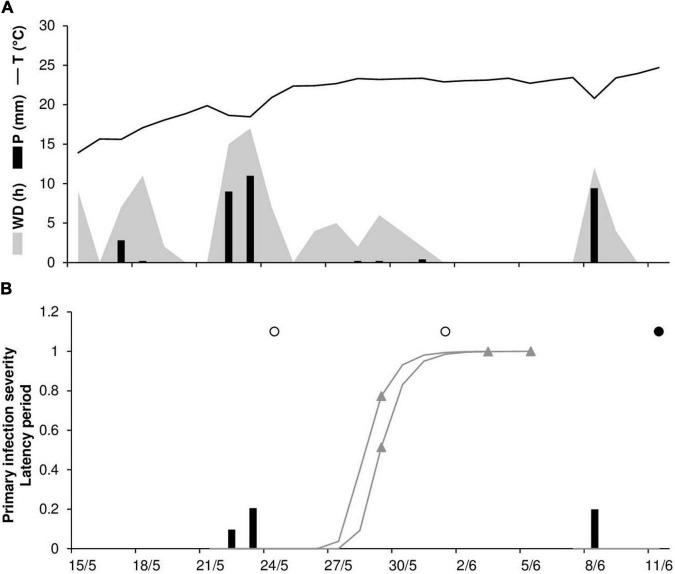
Predicted and observed primary infection onset in the experimental wheat field in Ravenna, Italy, in 2018 (RAV18). **(A)** Weather variables: air temperature (T, °C, solid line), rainfall (R, mm, black bars), and wetness duration (WD, in h, gray area). **(B)** Predicted primary infection (black bars); progress of latency (gray line); period of uredia eruption, from 50 to 100% of erupted uredia (line between triangles); days on which stem rust symptoms were not observed (empty dots); days on which uredia were observed in field plots (full dots).

As noted previously, there was one case (RAV18) in which the model did not predict a real infection and two cases in which the model provided a false positive prognosis (RAV17 and RAV18). At RAV18, the last day in which no stem rust symptoms were detected was June 1, and disease onset was recorded in the field on June 11. The false positive prediction was due to rain on May 22 and 23 that triggered model calculations for the infections, which reached 50% of uredia eruption on May 29 and 30 (Figure 3). Because the model assumes that *Pgt* uredospores are deposited on wheat tissues by rain ≥1 mm h^–1^, this single missed real infection may be attributable to rain events ≤1 mm h^–1^ that occurred at the end of May (Figure 3). As was the case for the false positive prediction at RAV18, the false positive prediction at RAV17 was due to a rain event that triggered model calculations. The predicted onset of disease occurred only 3 days before the last assessment in which no stem rust symptoms were detected (*not shown*).

### Evaluation of Disease Progress

**FIGURE 4:**
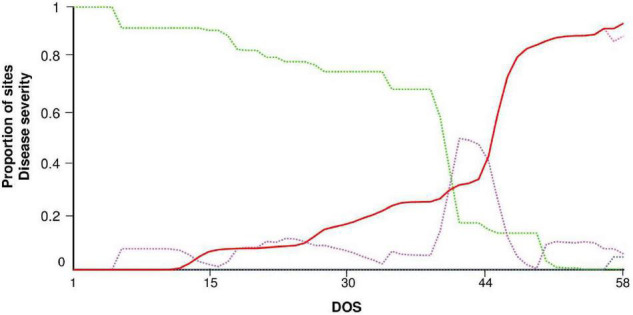
Model output for the experimental field in Rosemount, MN, United States, in 1968 (ROS68). Dynamics of sites: healthy (H, green dotted line), latent (L, purple dotted line), infectious (I, pink dotted line), removed (R, gray dotted line), and disease severity (DS, red solid line). Vertical axis: proportion of sites on a 0 to 1 scale. Horizontal axis: days of simulation (DOS); the model was run from June 5 (i.e., 9 days before the last disease assessment in which no stem rust symptoms were observed, see Table 3).

STELLA^®^ produced a dynamic representation of healthy (H), latent (L), infectious (I), and removed (R) sites. An example of model output for ROS68 is shown in Figure 4. The first seasonal infection occurred at day of simulation (DOS) 5. Repeated secondary infections triggered the increase of latent and infectious sites so that a rapid increase in disease severity (DS) was observed, especially after DOS 44. The exhaustion of infectious sites occurred only at the end of the epidemic, with a reduction of I and an increase of R.

**FIGURE 5:**
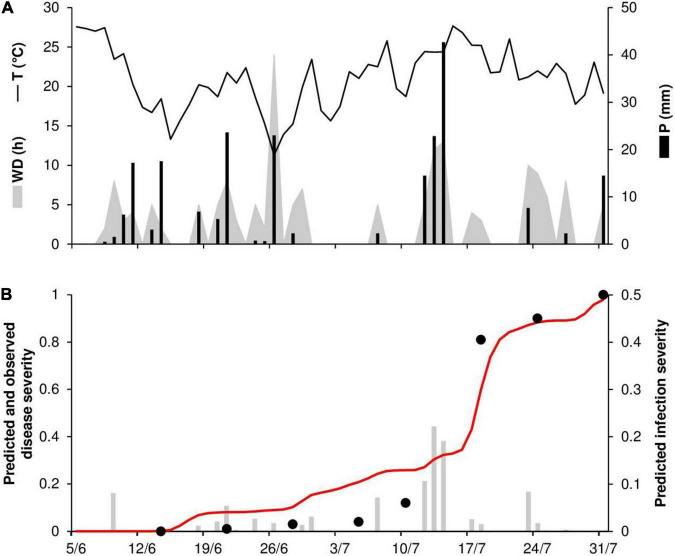
Predicted and observed stem rust progress in the experimental wheat field (susceptible cv. Purdue 5481C-1-13-2) in Rosemount, MN, United States, in 1968 (ROS68). **(A)** Weather variables: air temperature (T, °C, solid line), rainfall (R, mm, black bars), and wetness duration (WD, in h, gray area). **(B)** Infection severity predicted by the model (gray bars), disease severity predicted by the model (red line), and observed disease severity (full dots).

To evaluate the model’s ability to predict disease progress, predicted disease severity (DS) values were compared to observed disease severity values for six epidemics. At ROS68, the daily temperature ranged from 11°C to 28°C (mean = 21°C), with a total of 215 mm of rain on 20 rainy days and a total of 168 h of leaf wetness (Figure 5A). The disease was first recorded on June 21 and then increased quickly over time (especially between July 10 and 18), resulting in 100% disease severity on July 31 (Figure 5B). Regularly distributed infection periods ensured the progress of the disease, with a final predicted disease severity of 98% (Figure 5B). Goodness-of-fit of predicted versus observed data had a CCC = 0.959 and an RMSE = 0.11. A CRM = –0.043 indicated slight overestimation by the model.

**FIGURE 6:**
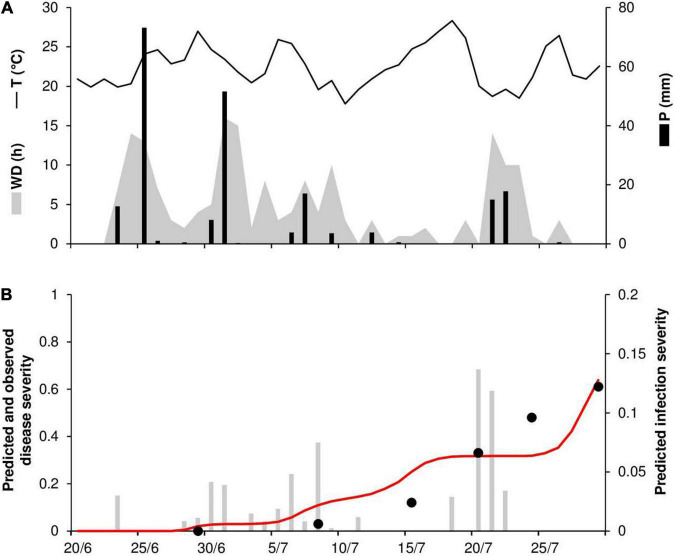
Predicted and observed stem rust progress in the experimental wheat field (susceptible cv. Prelude) in Rosemount, MN, United States, in 1978 (ROS78). **(A)** Weather variables: air temperature (T, °C, solid line), rainfall (R, mm, black bars), and wetness duration (WD, in h, gray area). **(B)** Infection severity predicted by the model (gray bars), disease severity predicted by the model (red line), and observed disease severity (full dots).

At ROS78, the daily temperature ranged from 18°C to 28°C (mean = 22°C), with a total 209 mm of rain on 15 rainy days and a total of 176 h of wetness (Figure 6A). Rains were frequent and intense at the end of June and in the first half of July, with prolonged wetness periods that led to the prediction of numerous infection periods (Figures 6A,B). Disease outbreak was recorded on July 8, and a rapid rise in the disease progress curve was observed, with a final disease severity of 61% (Figure 6B). Contrary to observations, the predicted disease progress was halted by a lack of wetness in mid-July that impeded the prediction of new infections, and it increased again only at the end of July, reaching 64% (Figure 6B). A CCC = 0.914 and an MRSE = 0.088 indicated good agreement between observed and predicted data; despite the failure of the model to predict the increase in disease in the second half of July, a CMR = –0.129 indicated a slight tendency toward overestimation.

**FIGURE 7:**
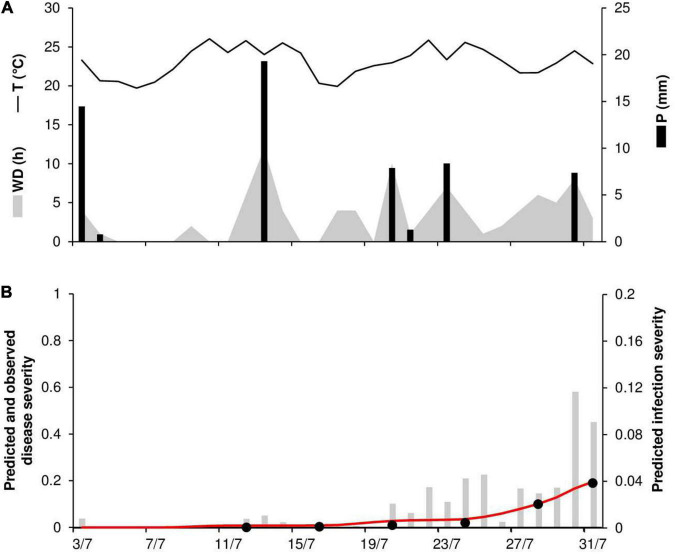
Predicted and observed stem rust progress in the experimental wheat field (susceptible cv. Prelude) in Rosemount, MN, United States, in 1979 (ROS79). **(A)** Weather variables: air temperature (T, °C, solid line), rainfall (R, mm, black bars), and wetness duration (WD, in h, gray area). **(B)** Infection severity predicted by the model (gray bars), disease severity predicted by the model (red line), and observed disease severity (full dots).

At ROS79, the daily temperature ranged from 20 to 26°C (mean = 23°C), with 59 mm of rain on 7 rainy days and 92 h of wetness (Figure 7A). Symptoms of stem rust were first observed on July 16; the disease progressed slowly until July 24 (disease severity 2%) but then rapidly increased until July 31, reaching 19% (Figure 7B). The model correctly predicted this dynamic, with a final severity of 19% (Figure 7B). High concordance of observed and predicted disease progress curves was obtained, with CCC = 0.988 and RMSE = 0.01. A CRM = –0.161 indicated a tendency of the model toward overestimation.

**FIGURE 8:**
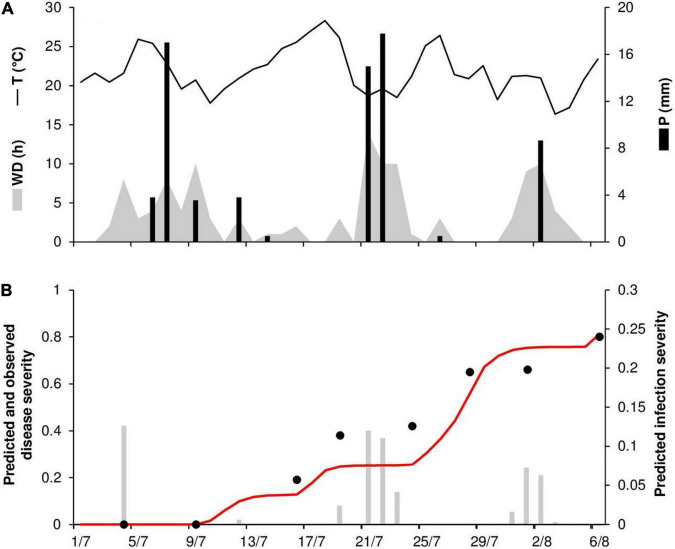
Predicted and observed stem rust progress in the experimental wheat field (susceptible cv. Prelude) in St. Paul, MN, United States, in 1978 (STP78). **(A)** Weather variables: air temperature (T, °C, solid line), rainfall (R, mm, black bars), and wetness duration (WD, in h, gray area). **(B)** Infection severity predicted by the model (gray bars), disease severity predicted by the model (red line), and observed disease severity (full dots).

At STP78, the daily temperature ranged from 16 to 28°C (mean = 22°C), with 71 mm of rain on 9 rainy days and 118 h of wetness (Figure 8A). Long wetness periods occurred between the second half of July and the beginning of August, resulting in several infections predicted by the model (Figure 8B). Disease onset was observed on July 7, and the epidemic was characterized by a substantial increase in disease, with a final severity of 80% (Figure 8B). The model predicted repeated secondary infections causing a substantial increase in disease, with a predicted final disease severity of 81%. For the goodness-of-fit of predicted versus observed data, the CCC = 0.953 and the RMSE = 0.091. The model showed a tendency toward underestimation (CRM = 0.112).

**FIGURE 9:**
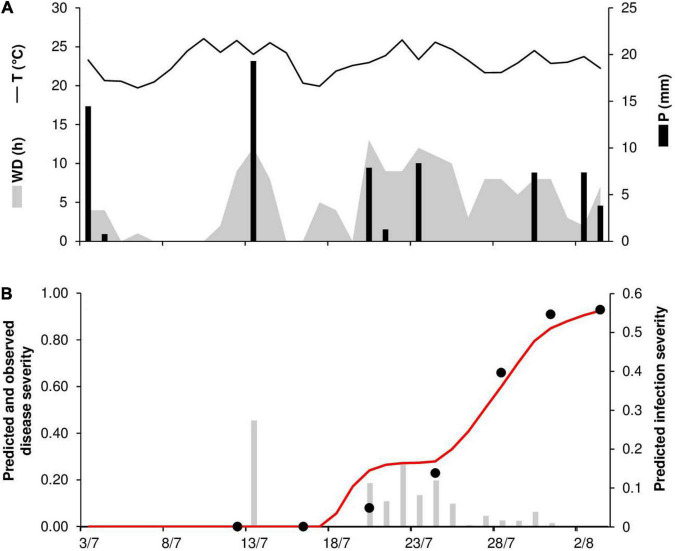
Predicted and observed stem rust progress in the experimental wheat field (susceptible cv. Prelude) in St. Paul, MN, United States, in 1979 (STP79). **(A)** Weather variables: air temperature (T, °C, solid line), rainfall (R, mm, black bars), and wetness duration (WD, in h, gray area). **(B)** Infection severity predicted by the model (gray bars), disease severity predicted by the model (red line), and observed disease severity (full dots).

At STP79, the daily temperature ranged from 20 to 26°C (mean = 23°C), with 55 mm of rain on 7 rainy days and 157 h of wetness (Figure 9A). Stem rust symptoms were first recorded on July 20. Long wetness periods occurred at the end of July, leading to a rapid increase of the epidemic and the prediction of repeated secondary infections by the model. Observed and predicted final disease severity was 93% (Figure 9B). Results showed good agreement between observed and predicted data, with CCC = 0.982 and RMSE = 0.071. A CRM = –0.03 indicated a slight tendency toward overestimation.

**FIGURE 10:**
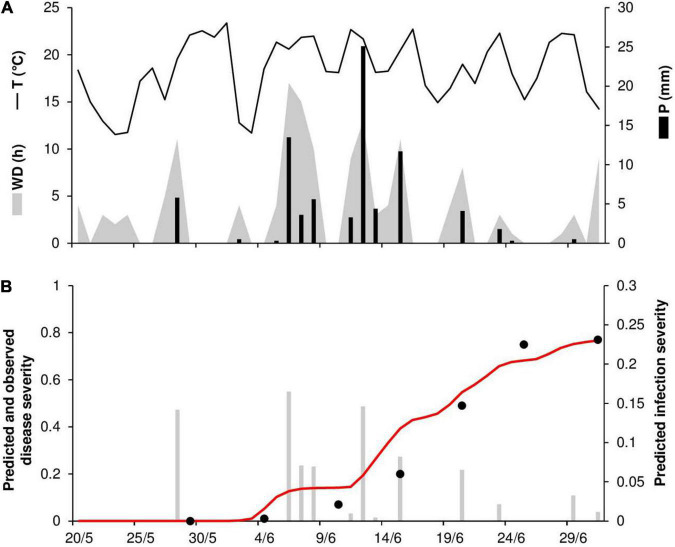
Predicted and observed stem rust progress in the experimental wheat field (susceptible cv. Tyler) in Rock Springs, PA, United States, in 1986 (PEN86). **(A)** Weather variables: air temperature (T, °C, solid line), rainfall (R, mm, black bars), and wetness duration (WD, in h, gray area). **(B)** Infection severity predicted by the model (gray bars), disease severity predicted by the model (red line), and observed disease severity (full dots).

At PEN86, the daily temperature ranged from 12 to 23°C (mean = 18°C), with 81 mm of rain on 14 rainy days and 147 h of wetness (Figure 10A). Disease outbreak occurred on June 4 and was followed by a rapid increase of the epidemic that resulted in a final disease severity of 77%, which was correctly predicted by the model (Figure 10B). Many secondary infections occurred in the first half of June, which was characterized by frequent rains and long wetness periods (Figure 10B). Goodness-of-fit of predicted versus observed data had CCC = 0.96, RMSE = 0.086, and CRM = –0.125.

**FIGURE 11:**
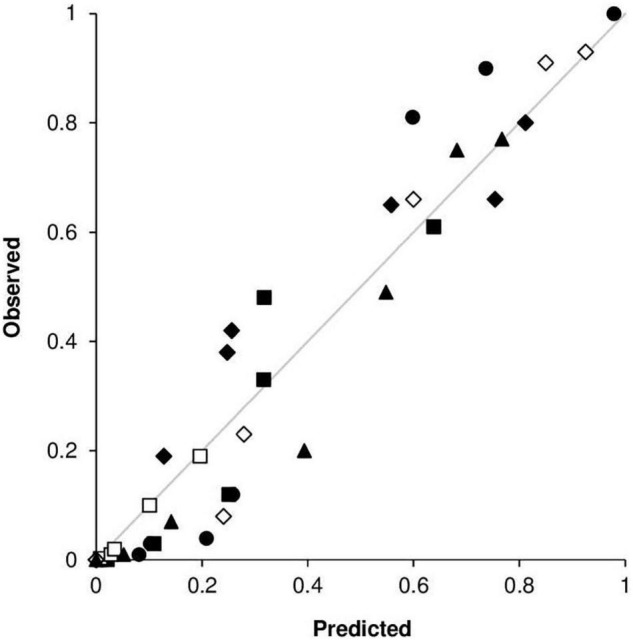
Predicted versus observed values of stem rust severity in the following locations and years: (•) Rosemount, MN, United States, in 1968; (■) Rosemount, MN, United States, in 1978; (□) Rosemount, MN, United States, in 1979; (◆) St. Paul, MN, United States, in 1978; (◆) St. Paul, MN, United States, in 1979; and (▲) Rock Springs, PA, United States, in 1986.

An overall comparison of predicted versus observed data (Figure 11) indicated a good agreement between the fitted line and the perfect agreement line (CCC = 0.96), with little average distance between real data and the fitted line (RMSE = 0.09). The model showed a slight tendency toward overestimation (CRM = –0.017).

## Discussion

The objective of this study was to develop a mechanistic model for stem rust of wheat by exploiting the available information on the pathogen and disease and by mobilizing that information *via* systems analysis (Leffelaar and Ferrari, 1989; Rossi et al., 2015). Unlike previous models of stem rust (Meyer et al., 2017; Mulatu et al., 2020; Willocquet et al., 2021), our model is predictive and mechanistic in that it considers the biological processes involved in the development of epidemics and the weather factors (i.e., rain, temperature, wetness duration, and light regime) affecting those processes.

The model focuses on uredospores, which are responsible for stem rust epidemics in temperate climates where the inoculum for the first infections in a season consists of air-transported uredospores that travel long distances from warmer areas (Hirst et al., 1967; Roelfs, 1985; Zadoks and Bouwman, 1985; Eversmeyer and Kramer, 2000). Eversmeyer and Kramer (2000) reported that airborne uredospores infecting winter and spring wheat on the Great Plains of the United States were likely produced in Mexico and southern Texas, and that the dissemination northward occurred in short hops of about 100 km. West and East European tracts of uredospore movement from North Africa to Grain Britain and Scandinavia, respectively, were described by Zadoks and Bouwman (1985) and Hirst et al. (1967). Although it was developed and tested in the temperate zone of Europe and North America, the current model should enable predictions in any area where wheat is grown.

The model’s core structure was adapted from the design of Zadoks (1971), which had been developed based on Vanderplank’s (1963) integro-differential equation for epidemics; in that equation, host sites go from healthy, to infected, to infectious, and finally to “removed” during the epidemic. This approach is a well-established modeling framework that has been used in several pathosystems and climates and for diseases of cereals and dicots (Savary et al., 1990, 2015; Djurle and Yuen, 1991; Rossi et al., 1997; Bove et al., 2020). The model’s structure was simple, leading to the assumptions that all sites have equal size that healthy sites have the same vulnerability and that diseased sites have a random distribution. Another simplification was that host growth, host senescence, lesion expansion, and disease management actions (e.g., plant resistance, crop management operations, or timing of crop establishment) were not considered. Because the model was validated against independent data, the model’s accurate predictions of stem rust epidemics indicate that these simplifications did not greatly reduce the model’s ability to make correct predictions. Moreover, the model validation was performed using data collected in a field planted with cultivars that were susceptible to stem rust, showing that the model can predict infection periods. The use of model predictions in crops planted with cultivars exhibiting lower levels of susceptibility to stem rust has to be evaluated. Nevertheless, the design of the model makes it easy to implement further modifications and improvements based on new scientific evidence. For instance, the use of less susceptible hosts can be easily addressed by incorporating modifiers (Loomis and Adams, 1983) accounting for resistance components, as previously implemented in similar model structures (Savary et al., 2012, 2015; Bove et al., 2021).

A daily time step was used for the main structure so that the rate of transfer (Rtrans) and rate of removal (Rrem) are calculated using the mean daily temperature. However, the use of daily weather data ignores the daily variations that may significantly affect infection and other epidemiological processes (Scherm and Van Bruggen, 1994). To avoid these inaccuracies, the model could be altered to assess infection rate (RI) as a function of hourly changes in temperature, wetness duration, and light regime. A time step of 1 h has been used to better account for the effect of fluctuating temperature and humidity, and of possible wetness interruptions during the day (Scherm and Van Bruggen, 1994; Narouei-Khandan et al., 2020).

Epidemics in the model begin on a given day (Onset), which depends on three weather factors (rain, wetness duration, and temperature) that are calculated hourly. As is the case for RI calculations, a time step of 1 h for Onset calculations ensures precise prediction of the first seasonal infection, based on the three conditions that initiate model calculations: (i) rain ≥1 mm h^–1^; (ii) wet period following rain ≥3 h; and (iii) mean temperature during wetness (Tw) 15 < Tw < 32°C. The modeled pattern of epidemic initiation is another element that required assumptions about uredospore deposition. Deposition of airborne uredospores that are transported for long distance is known to be triggered by scrubbing rainfall (Rowell and Romig, 1966; Nagarajan et al., 1977, 1976; Singh et al., 2006). Because an inability to quantify uredospore dose is implicit in the model, the model assumes that the uredospore dose ranges from 0 to 1 (INOCP; how the value of this parameter is selected is described later in the Discussion as are the associated limitations) and that a rain of at least 1 mm h^–1^ is able to cause uredospore deposition. This rain threshold was defined based on studies that related rain dynamics (duration and intensity) to spore concentration in the atmosphere (Rowell and Romig, 1966; Suffert, 1999). It is generally accepted that thunderstorms or rain showers usually require only a few minutes to remove the uredospores from the atmosphere (Eversmeyer et al., 1972; Suffert, 1999; Sache, 2000). Studies of rain duration (Suffert, 1999) showed that rapid scrubbing of airborne uredospores occurred during the first 10 min of a rain event. Rowell and Romig (1966) assessed spore concentrations in rain samples collected in the north-central United States during two seasons, and did not find any correlation between rain intensity and uredospores deposition; their data showed that uredospores were generally detected in rain samples of >1 mm.

The assumptions noted in the previous paragraph could result in false positive predictions of infection if no uredospores are deposited on the crop when predicted by the model, or could result in false negative predictions if uredospores are deposited on the crop when not predicted. Of the 30 cases used to validate the ability of the model to predict the first infection in the season, there were two cases in which the model predicted infections that were not observed (FPP = 0.091) and one case in which the model failed to predict infections that were observed (FNP = 0.125). Unjustified alarms (FPP) do not affect crop health but can lead to needless fungicide applications. To reduce this error, we require a better estimate of the presence of uredospores early in the season, i.e., better ways to estimate INOCP. Several authors (Rees, 1972; Nagarajan et al., 1976, 1977) proposed the use of spore traps or the sampling of rain water to monitor the presence of stem rust uredospores in the environment. The use of spore traps for monitoring airborne inoculum in support of epidemiological models has been suggested for other pathosystems (Rossi et al., 2007; Carisse et al., 2012).

False negative predictions (FNP) lead to missed real infections and reduce the model’s usefulness, because growers would fail to protect crops when necessary. This type of error occurred in only one case (at RAV18), and it was likely caused by a rain event of <1 mm h^–1^ or a dry deposition of uredospores. To avoid this error, we also considered reducing the rainfall threshold used by the model to predict uredospore deposition (we considered using ≥0.4 or 0.6 mm h^–1^), but this reduction led to an increase of FPP that significantly decreased the overall accuracy of the model (*not shown*). Further studies are needed on the effects of rain on the deposition of *Pgt* uredospores.

In this research, INOCP was estimated empirically: for each epidemic, different values of INOCP were initially considered and that value that resulted in the final disease severity closest to the real observation was selected and used for validation. This approach will not greatly affect the reliability of the model to predict disease progress because it modulates the final value of the disease severity but not its progress. Overall agreement of predicted and observed values was obtained, with high correlation between the fitted and prefect agreement line (CCC = 0.96) and little average distance between the real data and the fitted line (RMSE = 0.09), indicating good model accuracy (i.e., it provided predictions close to reality). Considering that the model was validated using independent data collected between 1968 and 1986 in the United States, further validation with recent data from sites with different climates (e.g., Mediterranean, temperate, and continental) should be performed to better evaluate the model’s robustness (i.e., its ability to provide accurate predictions in different environments and with different epidemiological conditions).

In spite of some shortcomings that mostly reflect its simplicity, the model seems to be useful for predicting epidemics of stem rust of wheat. In future, the model could be incorporated into existing decision support systems and used for scheduling fungicide applications to control the disease.

## Data Availability Statement

The raw data supporting the conclusions of this article will be made available by the authors, without undue reservation.

## Author Contributions

VR and IS mainly contributed to the conceptualization of the model. VR provided the methodology and the resources for the study. IS and FB implemented the model in STELLA^®^ and performed model validation. All authors contributed to the analysis of results, collaborated in writing the manuscript, contributed to the article, and approved the submitted version.

## Conflict of Interest

FB was employed by Horta Srl. The remaining authors declare that the research was conducted in the absence of any commercial or financial relationships that could be construed as a potential conflict of interest.

## Publisher’s Note

All claims expressed in this article are solely those of the authors and do not necessarily represent those of their affiliated organizations, or those of the publisher, the editors and the reviewers. Any product that may be evaluated in this article, or claim that may be made by its manufacturer, is not guaranteed or endorsed by the publisher.

## Footnotes

1
http://www.cabdirect.org

2
https://www.scopus.com

3
https://scholar.google.it

4
https://www.r-project.org
